# Myoelectric motor execution and sensory training to treat chronic pain and paralysis in a replanted arm: a case study

**DOI:** 10.1186/s12984-024-01508-5

**Published:** 2024-11-23

**Authors:** Morten B. Kristoffersen, Maria Munoz-Novoa, Mirka Buist, Mona Emadeldin, Carina Reinholdt, Max Ortiz-Catalan

**Affiliations:** 1 Center for Bionics and Pain Research, Mölndal, Sweden; 2 Department of Orthopaedics, Institute of Clinical Sciences, Sahlgrenska Academy, University of Gothenburg, Mölndal, Sweden; 3 Center for Advanced Reconstruction of Extremities (C.A.R.E.), Sahlgrenska University Hospital, Mölndal, Sweden; 4 Department of Hand Surgery, Institute of Clinical Sciences, Sahlgrenska Academy, University of Gothenburg, Sahlgrenska University Hospital, Gothenburg, Sweden; 5 Prometei Pain Rehabilitation Center, Vinnytsia, Ukraine; 6 Center for Complex Endoprosthetics, Osseointegration, and Bionics, Kyiv, Ukraine

**Keywords:** Replantation, Electromyography, Sensory feedback, Neurofeedback

## Abstract

**Background:**

Following upper limb amputation, surgeries such as arm transplantation or replantation might be an option to restore function. After such surgeries, rehabilitation of the arm is needed. However, conventional rehabilitation is dependent on some volitional movement of the arm. If there is no or minimal movement of the arm, conventional rehabilitation might not be successful. The purpose of this study is to evaluate a novel combination of myoelectric motor execution (MME) and sensory training (ST) to reduce pain and improve upper limb function in a person with a highly impaired replanted arm.

**Methods:**

The participant, a 72-year-old male, had his right arm replanted after a traumatic accident. No functional recovery was achieved following conventional rehabilitation and chronic neuropathic pain developed post-surgery. The participant then received 18 sessions of MME in which intended movements were decoded from the replanted arm’s myoelectric signals using machine learning and real-time feedback was provided on a screen. Nine sessions included ST using tactile grids where the participant discriminated different sensations.

**Results:**

The participant regained active extension of the thumb (4 degrees) and regained active wrist movement (flex: 6 degrees, extend: 10 degrees), both of which had no active movement prior the MME interventions. He also perceived an increase in sensation in the thumb and fingers. Pain levels fluctuated throughout the study and no consistent change could be concluded.

**Conclusion:**

MME is a novel virtual rehabilitation treatment which provides feedback using virtual limbs and serious games. MME combined with ST is a potential rehabilitation treatment for individuals with highly impaired arms and hands which might ameliorate chronic neuropathic pain.

## Introduction

After a major upper limb amputation, there are different options for restoring the function of the lost arm and hand. Traditionally, prosthetic devices can be fitted to the residual limb, but the abandonment rate is around 50% [1], and most prosthetic hands only offer one grasping function (single degree of freedom). An alternative solution is to replant the amputated limb. Replantation is possible if the amputated limb has not been severely crushed and not exposed to prolonged deprivation of oxygen and nutrients at body temperature (normothermic ischemia time) [2]. When a traumatic amputation has occurred, replantation should always be considered. An experienced hand surgeon with microsurgery skills can determine if replantation is feasible considering the prognosis of function, pain, and sensibility.

The benefit of limb replantation over a prosthesis is that sensory feedback, including proprioception, is possible and the replanted hand is usually more functional than a prosthetic one [2]. Furthermore, cosmesis is preserved with a replantation and there is no discomfort from wearing a prosthesis. Recent reviews found that patients who had the arm replanted were more satisfied and had higher patient reported outcomes, such as upper limb function, compared to patients who received a prosthesis [3, 4]. However, the limb replantation surgery is complex and post-surgical rehabilitation can take more than a year [5]. Furthermore, hand function and sensation is not guaranteed and since the nerves are severed and then surgically repaired, there is a risk that chronic neuropathic pain will develop [6]. Successful reattachment of the upper limb is possible in 77–93% of cases [7]. To the best of our knowledge, it is not known how many major limb replantations follows from major upper limb amputations.

Neuropathic pain is also common among individuals with upper limb loss. In this population, the pain is often referred to as phantom limb pain (PLP), which is pain felt in the lost limb. Similarly to the neuropathic pain experienced by arm replantation patients, the pain is often chronic and difficult to treat. To date, there is no treatment that effectively relieves PLP in the various existing cases, but a recent treatment called Phantom Motor Execution (PME) has shown promise in clinical trials [8, 9]. PME uses myoelectric pattern recognition to decode the movement of the phantom limb by classifying patterns of electromyographic activity from the residual limb. The output of the pattern recognition algorithm is used to control a virtual limb on a screen or to control video games. In a clinical trial of chronic intractable PLP, 14 participants for whom no other treatment was effective, underwent 12 sessions of PME and showed a reduction of pain of about 50%, which largely remained 6 months after the last treatment [9]. The cause for PLP is unknown, but several hypotheses exist [10]. The recent “stochastic entanglement” hypothesis for the neurogenesis of PLP is based on dynamical systems theory and states that severe impairment of the sensorimotor system can disrupt the healthy relationship between pain and somatosensory neural circuitries leading into a pathological state in which pain is processed despite the lack of noxious stimuli [8]. To relieve PLP thus requires that the affected limb neural circuitry (somatosensory and motor) is re-engaged in a purposeful manner and thereby disassociated from pain processing. Practically, this can be achieved by tasking the subject to produce movements with the affected limb and stimulating sensory feedback. The goal of PME is to re-engage the neural circuitry which was responsible for controlling the now amputated limb. We hypothesize that the origin of chronic pain caused by arm replantation is similar to that of PLP and can therefore be treated in an analogous manner.

Sensory training (ST) alone has been explored to alleviate PLP [11]. ST improves the participant’s ability to discriminate spatial and temporal somatosensory stimuli. As described in the preceding paragraph, the stochastic entanglement hypothesis suggests that motor and somatosensory neural circuitry can be engaged to alleviated pain, and fully engaging both would be the ideal treatment [8].

In this single-case design study, we combine a novel PME-like treatment, named Myoelectric Motor Execution (MME), with ST to treat chronic pain and restore function in a patient who had an arm replantation. The patient had his arm replanted at Sahlgrenska University Hospital, Sweden. After surgery, the patient reported pain which became chronic, and he had no function in the replanted hand and wrist. Following revision surgeries, the patient underwent extensive rehabilitation, but no improvement in pain or function were achieved. We therefore decided to investigate if MME and ST could alleviate pain and potentially improve the function in the paretic arm and hand. We hypothesize that MME can lead to improved motor function given that MME requires that the participant generates myoelectric signals (muscle contractions) with the muscles of the replanted arm. The generation of myoelectric signals infers that motor units are activated which leads to improved neural control. The real-time feedback provided by MME guides the participant to generate specific patterns of myoelectric activity, and said myoelectric activity helps to ensure that the participant is in fact attempting to perform actions.

The research questions of this study were directed to the possibility of using MME with ST applied as a supplement to (1) alleviate neuropathic chronic pain and (2) improve function of a highly impaired replanted arm.

## Methods

### Participant

The participant was a 72-year-old male who had his right arm amputated above the elbow joint due to trauma. Subsequently, the participant had the arm replanted. Following surgery, the participant had almost no sensation in the arm and poor hand and wrist function. To achieve grip function, a free gracilis muscle was surgically attached, but the vessels clotted, and the resulting wrist movement was limited. In a revision surgery, a tendon transfer of brachioradialis was performed. The brachioradialis was transferred to the wrist and a passive thumb by tenodesis of the flexor pollicis longus to the radius. The aim was to obtain better wrist and grip function. Osteoarthritis in the wrist was observed. The brachioradialis muscle was weak (Medical Research Counsil scale of 3) and the patient had to go through release of massive adhesions and tenolysis due to the previous operations. This resulted in a poor outcome of the last surgery, even though early active mobilization was done with the patient inhouse. Despite the replantation of the arm, the participant reported experiencing a phantom hand, which was probably caused by a lack of movement and somatosensory feedback. The participant’s phantom hand appeared occasionally, especially when he tried to move his replanted hand. Furthermore, his phantom arm was frozen including the wrist, but he could sometimes perform some phantom finger movements like a pinch. When the participant moved the phantom hand, he felt that the phantom hand became disassociated from the replanted hand, as the replanted hand had no active motion while the phantom hand was moving. Due to a combination of age, diabetes, and vascular disease, it was decided not to perform any additional revision surgeries. All surgeries were performed at Sahlgrenska University Hospital in Mölndal, Sweden.

Neuropathic pain emerged as a significant challenge following the initial replantation surgery. Comprehensive efforts were made to address it through pain management, physiotherapy, and conventional sensory training starting immediately after the surgery. The participant received a regimen including Gabapentin, Paracetamol, and Oxycontin to alleviate the pain. Despite undergoing approximately 30 training sessions of physiotherapy and occupational therapy in the first 12 weeks after surgery, functional recovery was not achieved. Additionally, the replanted arm occasionally experienced swelling, for which elastomull bandages were applied to provide compression and alleviate discomfort. The current study was approved by the Swedish Ethics Authority (Dnr.: 2020–07150). The participant was informed about the study and signed informed consent prior enrolment.

### Intervention

#### Training protocol

The treatment consisted of 20 sessions, aiming for a 10-week protocol with 2 sessions per week. Sessions 1 and 20 were assessment only, and no training was performed. Sessions 2 to 10 were MME only and sessions 11 to 19 consisted of MME and ST. In sessions 11 to 19, 75% of the time was spent on MME and the remaining time on ST. Sessions 6, 11 and 20 started with the monofilament test to assess sensory acuity (see outcome measures). Due to personal reasons, the participant was unable to adhere to the protocol, resulting in changes to the treatment frequency, with some weeks having only one or no sessions. There were 84 days between the first and the last session. Each session took approximately two hours including breaks.

#### Decoding of movement intent and myoelectric motor execution (MME)

To decode movement intent, eight channels of myoelectric signals were recorded at 1 kHz and transmitted wirelessly to a laptop computer using Neuromotus (Integrum AB, Sweden). The myoelectric signals were filtered and overlapping time windows with a length of 200 ms and 50 ms increment were extracted. The features mean absolute value, wavelength, zero crossings, and slope-sign changes were calculated for each window and used to train a linear discriminant analysis classifier. This method can decode movement intent in individuals who have almost no voluntary movement of the limb, as myoelectric activity precedes limb movement (cf. electromechanical delay [12]) and can therefore be used to predict movement intent even in the near absence of movement.

MME was based on PME [9, 13], but with MME the patient attempted to move the affected physical limb instead of a phantom limb. Electrodes were placed on the affected limb to record the attempted muscular contractions. The resulting myoelectric signals were analyzed using a pattern recognition algorithm that inferred the intended movement which was used to control a virtual limb or games. MME entails four steps:

1. The electrodes, up to eight pairs, are placed on the skin surface above the muscles in the affected limb.
2. The movements that are going to be trained are selected.
3. The participant performs a recording session, where the participant attempts to execute the selected movements while observing a virtual limb performing the same movement. Myoelectric signals are recorded during this process and used to train the pattern recognition classifier.
4. Training activities such as freeform virtual limb control, match testing, or gaming are performed with the participant in control. The quality of the control depends on the signals recorded during the recording session.

The participant started with movements such as pronation and supination, and flexion and extension of the wrist. Pronation and supination were trained specifically to activate and strengthen the brachioradialis muscle, which post-surgery was involved in extending the thumb. We aimed at restoring function of the thumb, to provide the participant with a key grip which would facilitate his daily life activities. In later sessions, the therapist guided the participant to train thumb extension with radial deviation performed bilaterally, which the participant was gradually able to perform. Only one degree of freedom (DoF) was performed at a time. The participant would sometimes perform movements bilaterally, to aid movement of his replanted arm. Moving the non-affected limb facilitates movement of the affected limb and therefore we tell participants to perform movements bilaterally when they struggle to execute movements with the affected limb. In the last sessions opening and closing of the hand was trained since the participant reported he could perform this movement with his phantom, but the participant was never able to fully close nor open the replanted hand.

#### Sensory training (ST)

For ST, we employed two tactile displays, each consisting of 16 tactors arranged in a 4 × 4 grid with an inter-tactor distance of one cm, strapped around the affected limb [14]. Each tactor can be pushed towards the skin, allowing for different stimulation patterns [15]. In addition, each grid can vibrate up to 250 Hz. During ST, the participant performs different activities:

- **Familiarization**: The participant was stimulated by different vibrotactile modalities without being asked to discriminate between them.
- **Sensory tasks**: In the sensory discrimination tasks, the participant was presented with different brief quizzes where the participant must discriminate between two types of stimuli. An example is “Fast or Slow” where the participant was given a slow vibrating stimulus, a fast-vibrating stimulus, and then a stimulus without label, and was then tasked with identifying whether the unlabeled stimuli was fast or slow. The participant could repeat the slow, fast, and unlabeled stimuli as many times as desired. Similar discrimination tasks exist for touch patterns, where an example could be that the participant must identify which column of tactors were activated.
- **Vibration game**: In the vibration game, the participant was presented with a vibration which must be identified on a scale from slow to fast of at least four possible vibrations. The participant could feel the target vibration and all the vibrations on the scale as many times as they want.
- **Memory Game**: In the memory game, the participant was presented with up to 12 cards depending on the difficulty level. The cards are presented face down and the participant must find the matching pair. Every time a card is turned, a sensation is felt (can be vibration or touch). When the participant turned two cards after each other which gave the same sensation, they were considered a match and were removed. The game ended when all matching pairs had been found. The participant in this study played the easy and medium difficulty levels with 8 and 12 cards respectively.

**Fig. 1:**
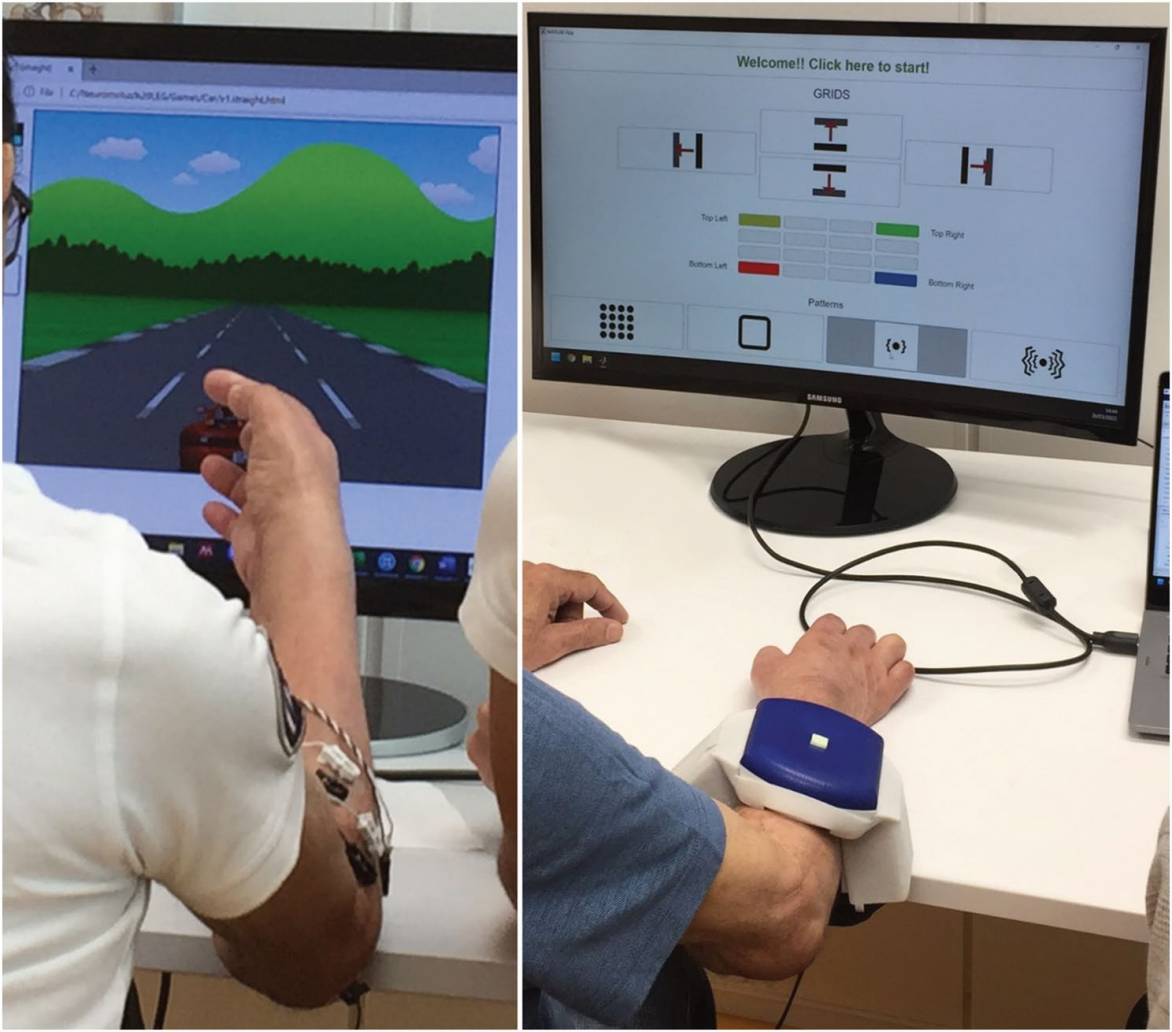
Left: The participant during MME playing a serious game. Right: The participant during sensory training

Initially, the tactile grids were placed below the elbow with a grid on the radial and ulnar side of the forearm (see Fig. 1). In later sessions, a grid was placed on the palmar side of the thumb. Since the participant reported pain from light touch (allodynia), there were concerns about ST being painful in the forearm. However, the participant did not perceive pain and found sensory stimuli provided by the tactile displays to be pleasant like a massage.

#### Outcome measures

For tracking changes related to pain the Questionnaire for Phantom Limb Pain (Q-PLP) was filled in after every session. Q-PLP was designed specifically to track phantom limb pain and was chosen as the patient reported some phantom phenomena [9, 13, 16]. Q-PLP includes the numeric rating scale (NRS) (scale none to maximum, 0 to 10), which is a common tool to assess pain at present. All questions related to prosthetic use or residual limb pain were skipped. The level of pain was also recorded using the Weighted Pain Distribution (WPD) [9, 13]. The WPD captures the percentage of time spent in six pain levels (scale none to excruciating, 0 to 5) by adding up six weighted portions with higher pain levels being weighted higher. Mathematically the WPD is calculated as:

$$WPD = \mathop \sum \limits_{p = 1}^5 p*{t_p}$$

where *p* indicates the pain level (0 to 5) and $\:{t}_{p}$ indicates the portion of time (0 to 1) in each pain level. The portion of time $\:{t}_{p}$ is calculated by dividing the hours in pain *p* between the hours awake.

At both assessment sessions and at session 10, the range of motion of flexion and extension of the elbow, forearm rotation, flexion and extension of the wrist, and flexion and extension of the thumb (metacarpophalangeal joint) was measured using a goniometer. For tracking changes in sensory perception, the Semmes-Weinstein monofilament test [17] was used in an exploratory fashion. The monofilament test is a test used for sensitivity testing and it consists of 20 probes which are designed to deliver the same force regardless of the experimenter. Commonly, the monofilament test starts by stimulating the participant with the smallest probe and increasing the probe size until the participant reports sensation in the stimulated location. However, the participant reported that the sensations were felt distant from the stimulated area, which is known as referred sensations. Due to the referred sensations, it was decided to continue to increase the probe size to see if it had an effect on the referred sensation, such as change of intensity or location. Continuing to increase the probe size after a sensation is reported is not how the monofilament test is commonly performed.

## Results

### Weighted pain distribution and numeric rating scale

**Fig. 2:**
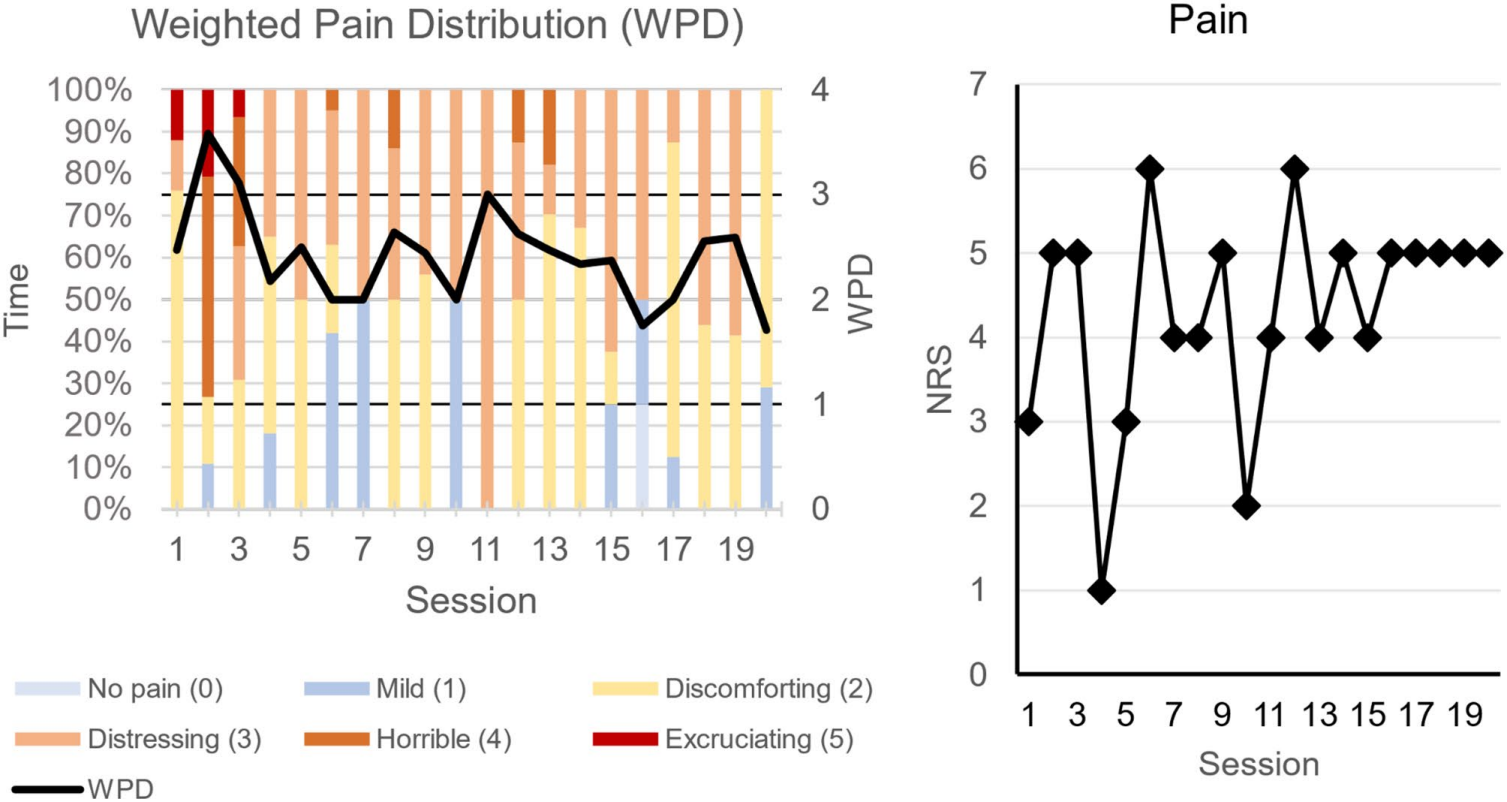
Left: Weighted Pain Distribution. Bars shows the percentage of time the participant was in different levels of pain as indicated by the color distribution of the bars. The WPD is plotted as a line. Right: Change in pain measured with the Numeric Rating Scale (NRS)

The Weighted Pain Distribution (WPD) fluctuated throughout the study and no conclusive decrease can be concluded. However, periods of the highest pain level disappeared after the third session and periods of the second to highest pain levels disappeared after session 12, which might be attributable to the intervention. See Fig. 2. The Numeric Rating Scale (NRS) of current pain fluctuated to a higher extent than the WPD and had not decreased at the final session. See Fig. 2. For both measures an initial increase was observed, a pattern that has also been observed with PME [13].

### Range of motion

**Fig. 3:**
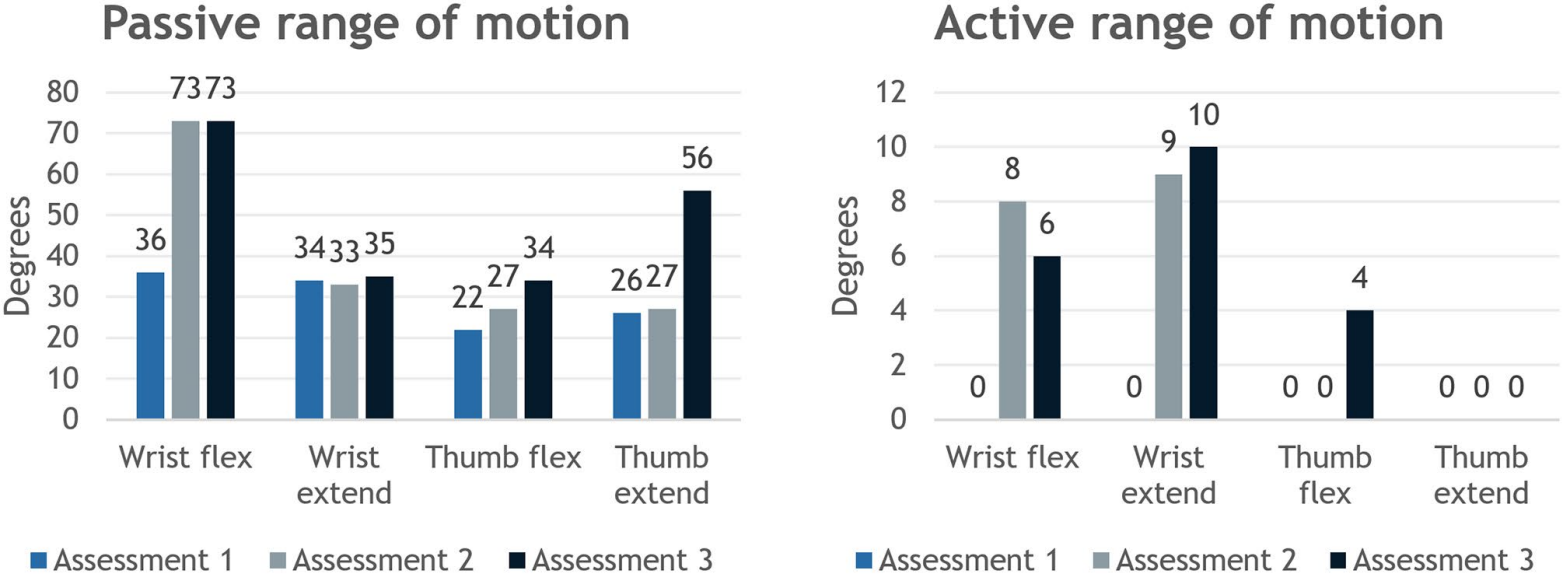
Left, passive range of motion for the wrist and thumb. Right, active range of motion for the wrist and thumb

ROM increased for wrist flexion and extension and for the thumb flexion and extension (metacarpophalangeal joint) of the affected arm. No consistent change was found for the remaining joints. Note that wrist flexion and extension and thumb flexion extension initially had no active motion but gained active motion at later sessions. Gained active ROM for the wrist was 6° flexion and 10° extension, and for the thumb 4° flexion was gained, see Fig. 3. For reference, the mean healthy active ROM for the wrist is 73–82° flexion and 59–75° extension depending on the study [18], and for the thumb it is approximately 60° flexion and 8.1° extension [19]. Note that the neutral position of the participant’s thumb was flexed, which is why the passive thumb extension extends beyond the healthy reference value. 

### Monofilament

**Fig. 4:**
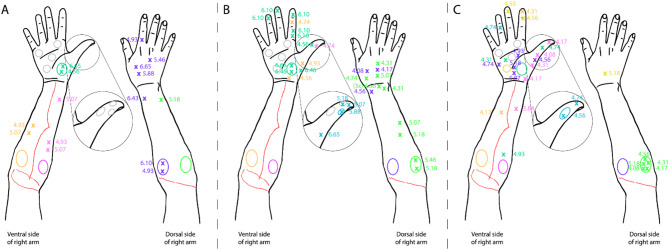
Raw data from the monofilament test from the affected arm from the ventral and dorsal side. Circles refer to the application site and crosses of the same color appertain to the location of the referred sensation from stimulation to that application site (e.g. green crosses represents the referred location from stimulation in the green circle). Numbers refer to monofilament thickness in millimeters. Grey circles refer to application sites which did not have a sensation. Data is from (**A**) session 6, (**B**) session 11 and (**C**) final assessment session. Note that no sensory training occurred between session 6 and 11

The results from the monofilament test are shown in Fig. 4; Table 1. Note how the application site and the location of the sensation vary widely (e.g. applications at the proximal part of the forearm were felt in the hand). There was considerable variability between the assessments. Referred sensations moved from the dorsal side of the hand to the ventral side in between assessments (e.g. purple area Fig. 4), and hardly any point from the first assessment was found in the last assessment. Stimulations in the green area were initially only felt in one location of the arm (Fig. 4A), but later also felt in multiple locations of the hand, arm, and stimulated area (Fig. 4B) and finally only in the stimulated area (Fig. 4C) meaning that referred sensations were replaced with expected sensations. Sensations also developed in the hand (e.g. yellow area) and thumb (i.e. blue area).

**Table 1:** Minimal detectable monofilament for each application site following the colors used in Fig. 4. NS = no sensation

	Session 6	Session 11	Session 20
Orange	4.93	4.56	4.17
Purple	4.93	4.08	4.56
Green	5.18	4.31	4.08
Pink	4.93	4.74	3.84
Petroleum green	4.56	5.46	4.31
Blue	NS	5.07	4.56
Yellow	NS	NS	4.56

However, the minimal detectable stimulation with a monofilament decreased for most sites which indicates that sensory perception improved during the treatment.

## Discussion

In this study, we found that for a patient with an arm replantation for whom conventional physio- and occupational therapy had no effect, MME resulted in partial improvement in wrist and thumb movements, which had no active movement. No clinically meaningful improvement in neuropathic pain was observed. One benefit of MME is the provision of real-time feedback, even in the absence of limb movement. Artificially presented biofeedback is especially important for participants who have little or no limb movement, as they receive limited somatosensory feedback from their own limb. Feedback might help these participants to become mindful of their limb and movement capabilities. MME is therefore especially suited for high impairments, such as the case presented in this study. In future studies, we aim to treat other sensorimotor impairments in larger samples and with appropriate control groups.

ST in this study was applied as a supplement to MME and the study was not designed to evaluate the efficacy of ST on its own. While the participant commented that he felt temporary alleviation directly after ST, the contribution of ST to the results cannot be established since no trend in pain measures was observed after its introduction in session 11. However, the participant did improve on the monofilament test and could detect smaller monofilaments after training. This suggest that ST improved sensory acuity, but not enough to impact pain or function in this particular case. We observed that probes which initially elicited no sensation later produced referred sensations, and that smaller probes also elicited referred sensations over time.

MME led to improved range of motion for the participant in this study, especially for the thumb which initially had no movement but was later mobile. However, the achieved range of motion was not sufficient for using the replanted arm in activities of daily living. Nevertheless, the improvements in range of motion might be sufficient for using an assistive device which can provide grip function. Grip function would presumably allow the participant to use the replanted arm in activities of daily living as a support for the non-affected arm.

MME supports the use of bilateral movements to facilitate movement of the affected limb and it has been shown that skills learned with one hand transfers to the other [20]. It is possible that the effects observed in this study can be explained by this phenomenon. For the participant reported in this study, where muscle tendons have been surgically moved, the motor control of the limbs differ since the muscles have different functions. Therefore, we find it unlikely that transfer effects can explain the results of this study. Finally, even if transfer effects contribute to the efficacy of MME, the feedback provided by MME is still important for participants to guide their rehabilitation.

The mechanism behind MME might be related to improved peripheral nerve regeneration. It has been found that motor execution improves peripheral nerve regeneration in rodent models [21, 22]. However, similar effects in humans have not been established. Given that MME facilitates targeted muscle activity, the persistent activation of compromised neural pathways might in turn facilitate peripheral nerve regeneration, especially in people with limited function. Future research should investigate the effect of MME on peripheral nerve regeneration.

In future work, we intend to make the implementation of MME intuitive and user friendly to such a degree that participants could conduct training by themselves after a short introduction. This will allow for rehabilitation for individuals with sensorimotor impairments in the home setting, which in turn might increase training exposure along with the possibility of long-term improvements. As many countries are faced with an ageing population [23] and thereby increasing the need for rehabilitation therapists, digitally-enabled therapies like the one presented here might reduce strain on healthcare resources.

The limitations of this study are the lack of qualitative assessments, the low adherence to the study protocol, the limited assessments before, during, and after the proposed intervention, and the inherent limitations of a study with one participant such as limited data and lack of a control.

## Data Availability

No datasets were generated or analysed during the current study.
